# Performance Comparison of Three Rapid Tests for the Diagnosis of Drug-Resistant Tuberculosis

**DOI:** 10.1371/journal.pone.0136861

**Published:** 2015-08-31

**Authors:** Antonino Catanzaro, Timothy C. Rodwell, Donald G. Catanzaro, Richard S. Garfein, Roberta L. Jackson, Marva Seifert, Sophia B. Georghiou, Andre Trollip, Erik Groessl, Naomi Hillery, Valeriu Crudu, Thomas C. Victor, Camilla Rodrigues, Grace Shou-Yean Lin, Faramarz Valafar, Edward Desmond, Kathleen Eisenach

**Affiliations:** 1 University of California San Diego, La Jolla, California, United States of America; 2 University of Arkansas, Fayetteville, Arkansas, United States of America; 3 Stellenbosch University, Stellenbosch, South Africa; 4 Institute of Phthisiopneumology, Chisinau, Moldova; 5 PD Hinduja Hospital and Medical Research Centre, Mumbai, India; 6 Microbial Diseases Laboratory, California Department of Public Health, Richmond, California, United States of America; 7 San Diego State University, San Diego, California, United States of America; 8 University of Arkansas, Little Rock, Arkansas, United States of America; The Catholic University of the Sacred Heart, Rome, ITALY

## Abstract

**Background:**

The aim of this study was to compare the performance of several recently developed assays for the detection of multi- and extensively drug-resistant tuberculosis (M/XDR-TB) in a large, multinational field trial.

**Methods:**

Samples from 1,128 M/XDR-TB suspects were examined by Line Probe Assay (LPA), Pyrosequencing (PSQ), and Microscopic Observation of Drug Susceptibility (MODS) and compared to the BACTEC MGIT960 reference standard to detect M/XDR-TB directly from patient sputum samples collected at TB clinics in India, Moldova, and South Africa.

**Results:**

Specificity for all three assays was excellent: 97–100% for isoniazid (INH), rifampin (RIF), moxifloxacin (MOX) and ofloxacin (OFX) and 99–100% for amikacin (AMK), capreomycin (CAP) and kanamycin (KAN) resistance. Sensitivities were lower, but still very good: 94–100% for INH, RIF, MOX and OFX, and 84–90% for AMK and CAP, but only 48–62% for KAN. In terms of agreement, statistically significant differences were only found for detection of RIF (MODS outperformed PSQ) and KAN (MODS outperformed LPA and PSQ) resistance. Mean time-to-result was 1.1 days for LPA and PSQ, 14.3 days for MODS, and 24.7 days for MGIT.

**Conclusions:**

All three rapid assays evaluated provide clinicians with timely detection of resistance to the drugs tested; with molecular results available one day following laboratory receipt of samples. In particular, the very high specificity seen for detection of drug resistance means that clinicians can use the results of these rapid tests to avoid the use of toxic drugs to which the infecting organism is resistant and develop treatment regiments that have a higher likelihood of yielding a successful outcome.

## Introduction

In 2013, the World Health Organization (WHO) estimated that less than half of all drug-resistant tuberculosis (DR-TB) cases were detected globally [1]. Undetected resistance results in treatment failure and increases the risk of TB transmission and patient mortality [2–4]. The lack of available, globally approved diagnostic platforms capable of rapidly detecting multi- and extensively drug-resistant TB (M/XDR-TB) remains a critical gap. MDR-TB is defined as TB resistant to both isoniazid (INH) and rifampin (RIF) while XDR-TB is MDR-TB with additional resistance to any fluoroquinolone, including moxifloxacin (MOX) or ofloxacin (OFX), and one or more of the injectables: amikacin (AMK), kanamycin (KAN), or capreomycin (CAP).

Recognizing that rapid, accurate drug susceptibility testing (DST) is critical for effectively treating and halting transmission of DR-TB [5, 6], clinical and laboratory partners in India, Moldova, the Philippines, Peru, South Africa, and the United States formed the Global Consortium for Drug-resistant TB Diagnostics (GCDD, S1 Text) to develop, test, and improve rapid TB diagnostic assays for M/XDR-TB [7–10].

Line Probe Assays (LPAs) MTBDR*plus* and MTBDR*sl* (Hain Lifescience, Nehren, Germany) detect resistance to INH, RIF, MOX, OFX, AMK, KAN, CAP and ethambutol through DNA amplification and oligonucleotide probe hybridization to resistance-conferring regions of the *Mtb* genome [11]. While version 1 of MTBDR*plus* was recommended by the WHO for the diagnosis of MDR-TB in 2008, the recently released version 2 has not been extensively studied [12,13]. The MTBDR*sl* LPA underwent WHO Expert Panel Review in 2013, but was not recommended as a replacement test for phenotypic DST for lack of sufficient performance data [14]. Pyrosequencing (PSQ) is a rapid sequencing technique used in several commercially available platforms; however, it is still under development for use as a TB diagnostic [8,15,16]. Our noncommercial PSQ M/XDR-TB assay involves sequencing and interpreting targeted, resistance-conferring regions of the *Mtb* genome. PSQ has shown high sensitivity and specificity for detection of M/XDR-TB isolates in controlled laboratory settings, but has not been evaluated in a large clinical trial [17,18]. Microscopic Observation of Drug Susceptibility (MODS) is a noncommercial assay where drug-containing wells are inoculated directly with concentrated sputum and subsequent *Mtb* growth is observed microscopically [19]. MODS has demonstrated high sensitivity and specificity for INH and RIF [20]. This study is the first large-scale clinical evaluation of extended MODS for XDR-TB.

## Methods

### Overview

Following Standards for the Reporting of Diagnostic Accuracy Studies (STARD), we completed a multicenter, prospective, head-to-head clinical evaluation study to compare the performance and speed of LPA, PSQ, and MODS for diagnosing drug resistance in patients at risk for M/XDR-TB, relative to the WHO approved phenotypic reference standard of MGIT DST [21,22].

Clinical sites for the study included P.D. Hinduja National Hospital in Mumbai, India, the Phthisiopneumology Institute in Chisinau, Moldova, and multiple primary care clinics in Port Elizabeth, South Africa in collaboration with Stellenbosch University. Patients were treated at both public and private hospitals and clinics depending on study site. Our study, registered with ClinicalTrials.gov (#NCT02170441), was reviewed and approved by institutional review boards at University of California, San Diego and each of the study sites. All participants provided written informed consent. Participation did not alter the standard of care. To ensure procedures were standardized across all sites, a comprehensive validation process for laboratory and data collection procedures was completed prior to enrollment at all sites [7].

### Enrollment and Sputum Collection

Patients presenting with risk factors for drug-resistant TB, who were known to be acid-fast bacilli (AFB) smear positive, or GeneXpert *Mtb* positive within the prior 14 days, and were age five or older were invited to participate. Frequency and patterns of recruitment were sequential but allowed to vary by site to accommodate individual site capacity. Two samples, one collected at enrollment and a second collected by the participant the following morning, were pooled and used for testing after decontamination and concentration by NALC-NaOH method [23].

### Standard Drug Susceptibility Testing (DST)

DST for all *Mtb* culture positive specimens were performed with the MGIT system using manufacturer recommended methods and published WHO-recommended critical drug concentrations: INH 0.1, RIF 1.0, MOX 0.25, OFX 2.0, AMK 1.0, KAN 2.5, and CAP 2.5 (μg/ml) [24,25].

### Rapid Diagnostic Assays

The LPAs used for this study were the GenoType MTBDR*plus* version 2 for detecting INH and RIF resistance and the GenoType MTBDR*sl* version 1 for MOX, OFX, AMK, KAN, and CAP resistance, performed following Hain manufacturer instructions [26,27]. LPA assays were performed on the sputum samples after they were decontaminated and concentrated as described above and were not repeated if results were uninterpretable or indeterminate.

The PSQ platform and methods used for this study have been detailed previously [8]. Briefly, PCR was performed to amplify targeted sequences using DNA extracted from decontaminated and concentrated sputum; PyroMark Q96ID system was used to generate sequencing data. Then, PyroMark IdentiFire software was used to align generated sequences to our library of wild type and mutant sequences. The gene target IS*6110* identified *Mtb*, while targets used for detection of drug resistance included: *katG*, *inhA* promoter, and *ahpC* promoter for INH; *rpoB* for RIF; *gyrA* for MOX and OFX; and *rrs* for AMK, KAN, and CAP [8,28]. If PSQ failed for one or more targets for any isolate, those targets were repeated in duplicate. Results of the repeated reactions were considered only if one or both of the results yielded a 100% match with the sequencing library. If initial failures did not resolve upon re-testing, PSQ results were considered indeterminate.

The MODS test was performed as previously described using standard drug concentrations: INH 0.4, RIF 1.0, MOX 0.5, OFX 1.0, AMK 2.0, and KAN 5.0 (μg/ml) [10,29]. Preliminary studies did not result in a decisive critical concentration for CAP, so multiple concentrations, 1.25, 2.5, 5.0, and 10.0 (μg/ml), were used in the study, but a critical concentration of 2.5 μg/ml was used for this analysis [10]. The MODS test was not repeated in the event of indeterminate results since when the indeterminate result was observed the original specimen was no longer available.

Methods of data monitoring, reporting, and collation for this study have been described previously [7]. The supporting information files contain the optimal data set underlying the findings of this study, with all additional data available upon request.

### Statistical Analysis

Using MGIT DST as a reference standard, sensitivity, specificity, positive/negative predictive values, and positive/negative likelihood ratios were calculated for each drug and rapid diagnostic test combination that produced interpretable results.

Discrepant cases between the MGIT reference standard and the three rapid assays were statistically tested using the Cochran's Q test which tests for differences in binary responses between three or more matched sets of frequencies or proportions [30]. To investigate the interpretability of the rapid test results (with respect to MGIT reference standard), each participant's rapid assay results were coded 'success' (when the rapid test read Resistant or Susceptible and so did MGIT) or 'failure' (when the rapid test read Failure/Indeterminate and MGIT was Resistant or Susceptible). If significant differences (p<0.05) were found, at least one rapid test had a different proportion of interpretable results than the others (with respect to the MGIT standard) thus we performed a post-hoc pairwise McNemar's test to identify the nature of the difference.

To investigate the accuracy of rapid test results (with respect to MGIT reference standard), each participants rapid assay results were coded 'success' (when the rapid test read Resistant and so did the MGIT or the rapid test read Susceptible and so did MGIT) or 'failure' (when the rapid test disagreed with MGIT). If significant differences (p<0.05) were found, at least one rapid test had a different accuracy than the others (with respect to the MGIT standard) thus we performed a post-hoc pairwise McNemar's test to identify the nature of the difference.

Time-to-results (TTR) was examined for all assays that produced interpretable results. For the growth-based assays (MGIT and MODS), TTR was based on the entire period from sample processing through test completion. For the DNA-based assays (LPA and PSQ), TTR was calculated based on time for sample processing through DNA extraction and then PCR/test set up through completion, excluding time DNA was stored for batching.

## Results

### Patients

**Fig 1:**
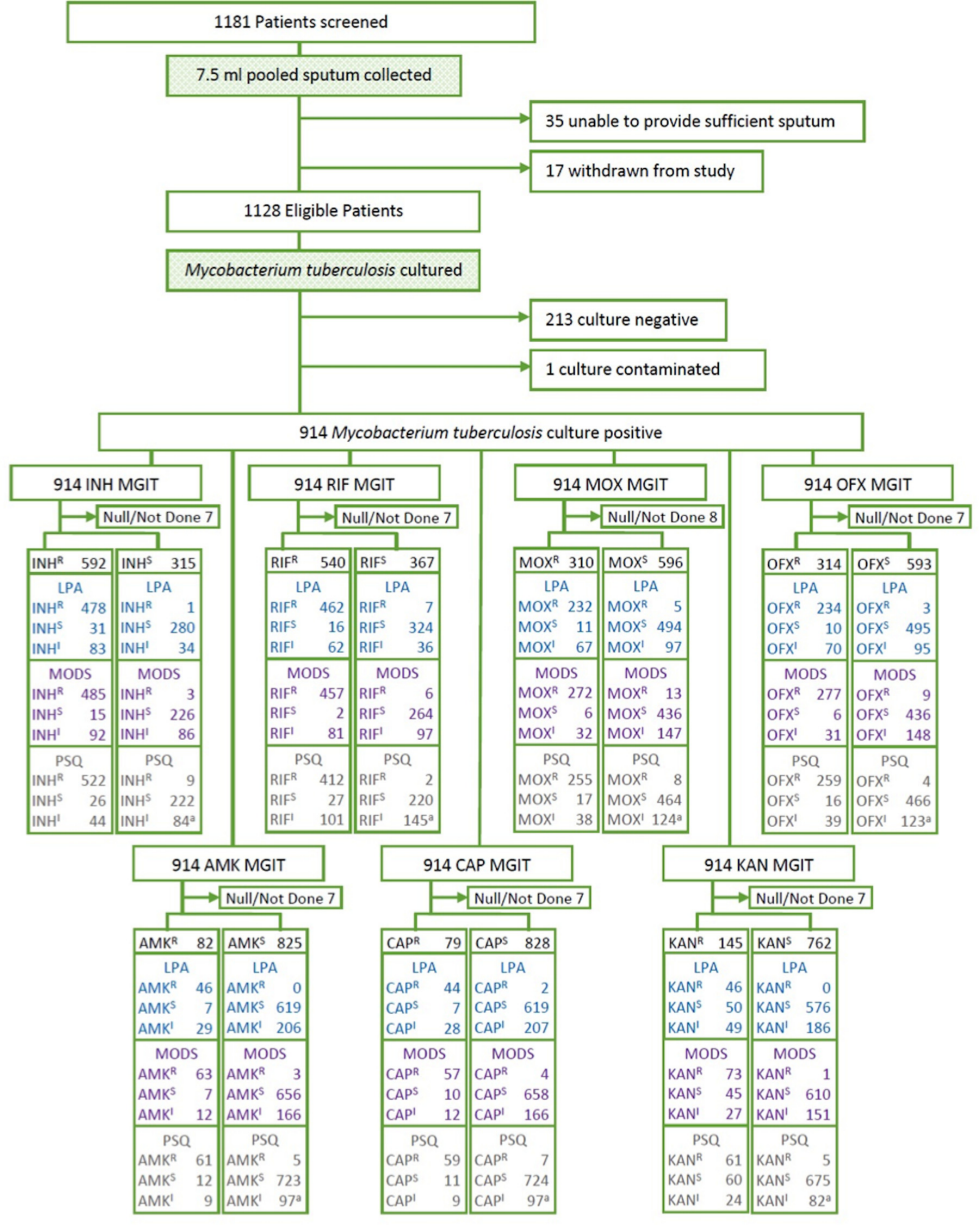
Flowchart of patient enrollment, clinical sample processing for Line Probe Assay (LPA), Microscopic Observation of Drug Susceptibility (MODS), and Pyrosequencing (PSQ) for seven anti-TB drugs: isoniazid (INH), rifampin (RIF), moxifloxacin (MOX), ofloxacin (OFX), amikacin (AMK), kanamycin (KAN), and capreomycin (CAP). Includes one null PSQ sample result (denoted as 'a'). R: Resistant, S: Susceptible, and I: Indeterminate.

A total of 1181 patients were screened for the study; one participant was determined to be ineligible and 52 were withdrawn (mostly due to inability to produce sufficient sputum), resulting in a total of 1128 study participants (612 from India, 254 from Moldova, and 262 from South Africa). While all study participants were smear positive during the 14 days previous to enrollment, only 73% (826) of pooled sputa collected at enrollment were smear positive and 81% (914) of participants were culture positive for *Mtb* (Fig 1). The mean age of participants was 34 years (range: 8–79 years) and gender skewed male (64% vs. 36%) (Table 1). Most participants (74%) received at least one month of prior TB treatment and 47% were clinically failing treatment at time of enrollment. HIV test results were available for 73% of enrolled participants, and 19% of these were positive. South Africa had the highest rates of participants receiving >1 month of treatment for a prior TB episode (97%), and the highest rates of HIV positive participants (54%). Participants from Moldova had the highest rate of close contacts with known drug-resistant TB cases (78%). Thirty-three percent of the study population (n = 305) were susceptible to all seven study drugs, and half (50%) had MDR-TB. India had the highest proportion of participants with MDR-TB (68%) and XDR-TB (11%).

**Table 1:** Demographic, Clinical and Laboratory Characteristics of the Patients.

Variable	Value	India	Moldova	South Africa	Total
Number of patients—n		612	254	262	1128
Age, years—median (range)		28 (8–78)	41 (12–79)	38 (13–66)	34 (8–79)
Gender—n (%)	Male	329 (53∙8)	205 (80∙7)	183 (69∙9)	717 (63∙6)
	Female	283 (46∙2)	49 (19∙3)	79 (30∙1)	411 (36∙4)
Ethnicity—n (%)	Hispanic	1 (0∙2)	1 (0∙4)	-	2 (0∙2)
	Non-Hispanic	611 (99∙8)	253 (99∙6)	262 (100)	1126 (99∙8)
Race—n (%)	White	-	254 (100)	2 (0∙8)	256 (22∙7)
	Black	-	-	260 (99∙2)	260 (23∙0)
	Asian/Indian	612 (100)	-	-	612 (54∙3)
	Pacific Islander	-	-	-	-
	Native American	-	-	-	-
	Other	-	-	-	-
***Screening Criteria***					
AFB smear+ within 14 days—n (%)	Yes	612 (100)	254 (100)	262 (100)	1128 (100)
	No	-	-	-	-
a) Previously received >1 month of treatment for a prior TB episode—n (%)	Yes	526 (86∙0)	59 (23∙2)	254 (97∙0)	839 (74∙4)
	No	38 (6∙2)	192 (75∙6)	8 (3∙0)	238 (21∙1)
	Unknown	48 (7∙8)	3 (1∙2)	-	51 (4∙5)
b) Failing standard TB treatment—n (%)	Yes	487 (79∙6)	16 (6∙3)	25 (9∙5)	528 (46∙8)
	No	112 (18∙3)	236 (92∙9)	234 (89∙3)	582 (51∙6)
	Unknown	13 (2∙1)	2 (0∙8)	3 (1∙2)	18 (1∙6)
c) Close contact with known drug-resistant TB case—n (%)	Yes	169 (27∙6)	197 (77∙5)	29 (11∙1)	395 (35∙0)
	No	208 (34∙0)	51 (20∙1)	167 (63∙7)	426 (37∙8)
	Unknown	235 (38∙4)	6 (2∙4)	66 (25∙2)	307 (27∙2)
d) Diagnosis with MDR-TB within last 30 days—n (%)	Yes	141 (23∙0)	70 (27∙6)	26 (9∙9)	237 (21∙0)
	No	398 (65∙0)	184 (72∙4)	236 (90∙1)	818 (72∙5)
	Unknown	73 (12∙0)	-	-	73 (6∙5)
e) Previously diagnosed with MDR-TB and are failing TB treatment	Yes	240 (39∙2)	9 (3∙5)	-	249 (22∙1)
	No	179 (29∙2)	245 (96∙5)	262 (100)	686 (60∙8)
	Unknown	193 (31∙5)	-	-	193 (17∙1)
***Patient Characteristics***					
Previous treatment for TB—n (%)	Yes	312 (51∙0)	60 (23∙6)	248 (94∙6)	620 (55∙0)
	No	156 (25∙5)	193 (76∙0)	13 (5∙0)	362 (32∙1)
	Unknown	144 (23∙5)	1 (0∙4)	1 (0∙4)	146 (12∙9)
If yes, number of prior TB episodes—mean, median (range)		1∙1,1 (0–5)	1∙4,1 (1–4)	2∙3,2 (0–10)	1∙6,2 (0–10)
Body Mass Index (BMI)–mean, median (range)		17∙9,17∙5 (9∙0–32∙9)	20∙4,19∙9 (12∙0–37∙2)	18∙4,17∙9 (7∙0–32∙4)	18∙6,18∙3 (7∙0–37∙2)
HIV Status—n (%)	Positive	14 (2∙3)	2 (0∙8)	141 (53∙8)	157 (13∙9)
	Negative	310 (50∙6)	252 (99∙2)	105 (40∙1)	667 (59∙1)
	Unavailable	288 (47∙1)	-	16 (6∙1)	304 (27∙0)
***Laboratory Results***					
Standardized Results—n (%)	Smear +	511 (83∙5)	171 (67∙3)	144 (55∙0)	826 (73∙2)
	Culture Positive	492 (80∙4)	226 (89∙0)	196 (74∙8)	914 (81∙0)
Standardized DST—n (%)	Pan Susceptible	75 (15∙2)	87 (38∙2)	143 (73∙3)	305 (33∙3)
	Mono INH^R^	19 (3∙9)	16 (7∙0)	16 (8∙2)	51 (5∙6)
	Mono RIF^R^	2 (0∙4)	-	3 (1∙5)	5 (0∙5)
	MDR-TB	335 (68∙1)	102 (44∙7)	17 (8∙7)	454 (49∙6)
	XDR-TB	54 (11∙0)	14 (6∙1)	12 (6∙2)	80 (8∙7)

### Sensitivity and Specificity

**Fig 2:**
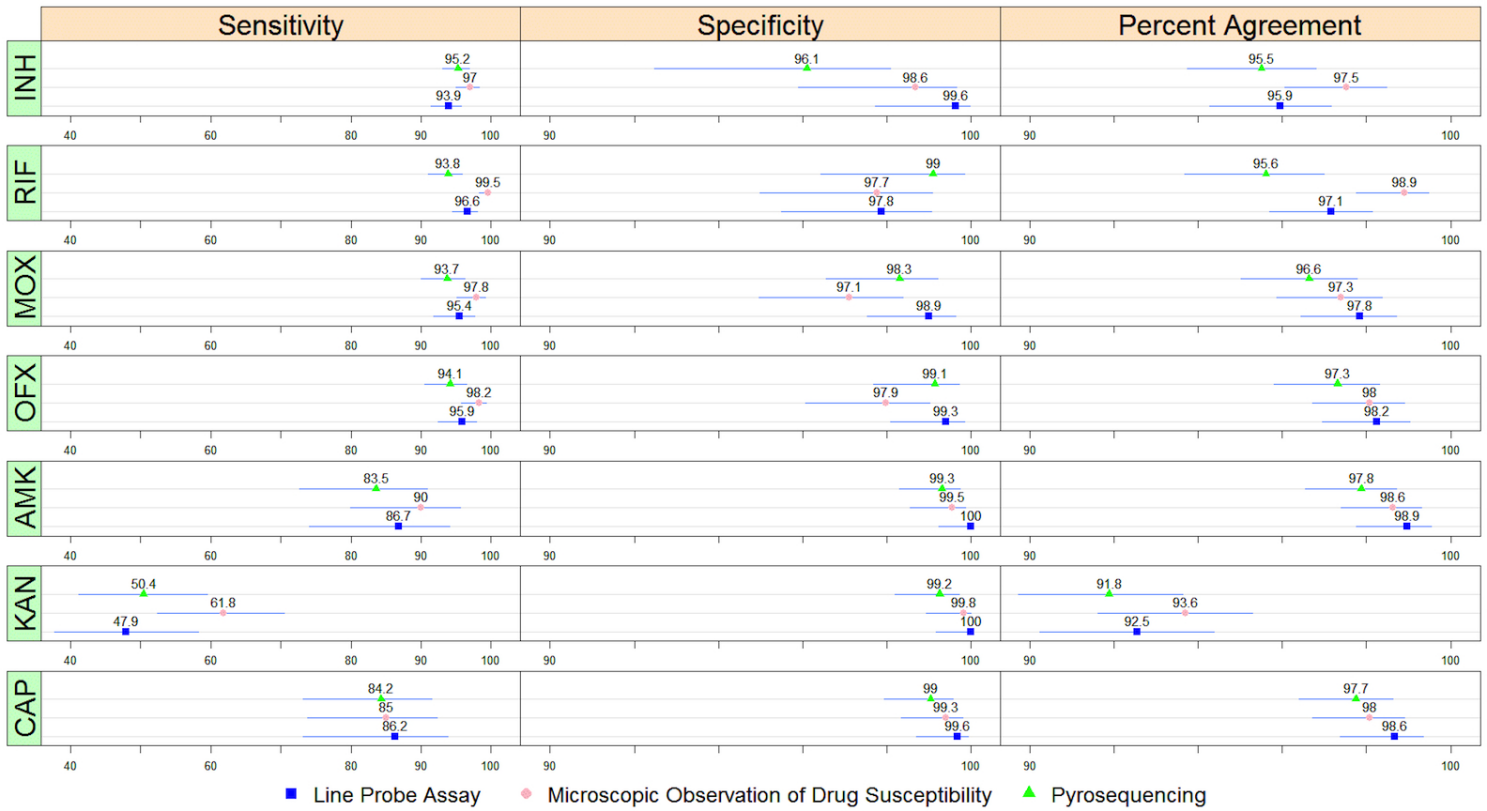
Sensitivity, specificity and percent agreement between three rapid drug susceptibility tests and MGIT for seven drugs: isoniazid (INH), rifampin (RIF), moxifloxacin (MOX), ofloxacin (OFX), amikacin (AMK), kanamycin (KAN), and capreomycin (CAP). Mean values and 95% confidence intervals for each comparison are represented in the figure; full data is included as a supplementary table.

All three assays performed well for direct detection of INH and RIF resistance, with specificities ranging from 96% to 100% and sensitivities ranging from 94% to 100% (Fig 2, S1 Table). For direct detection of MOX and OFX resistance, specificities and sensitivities were high, 94% to 99% and 94% to 98%, respectively. For the detection of resistance to AMK or CAP specificity was also very high (almost 100%) while sensitivity was considerably lower (84%-90% for AMK and CAP and 48%-68% for KAN). The positive likelihood ratios of all three assays for all seven drugs were >20 and negative likelihood ratios were all ~0.1 or lower, with the exception of the assays detecting KAN resistance, which had somewhat higher negative likelihood ratios (0.38–0.52).

### Comparative Assay Performance

The Cochran's Q test demonstrated significant differences in the three tests' ability to yield interpretable results for INH, RIF, AMK, KAN, and CAP, but not for OFX and MOX (Table 2 and S1 Table). For INH, LPA, and PSQ yielded significantly more interpretable results than MODS, while for RIF, LPA yielded significantly more interpretable results than MODS, which yielded significantly more interpretable results than PSQ. For the injectables (AMK, KAN, and CAP) PSQ yielded significantly more interpretable results than MODS, which yielded significantly more interpretable results than LPA (Table 2).

**Table 2:** Comparison of the ability of three diagnostic assays to produce interpretable and results agreeing with MGIT drug susceptibility testing for isoniazid (INH), rifampin (RIF), moxifloxacin (MOX), ofloxacin (OFX), amikacin (AMK), kanamycin (KAN), and capreomycin (CAP).

Drug	Interpretable Results		Agreement with MGIT DST	
	Overall Difference^a^	Pairwise Difference^b^	Overall Difference	Pairwise Difference
**INH**	χ^2^ = 28∙3	LPA*** & PSQ** > MODS	χ^2^ = 0∙70	N/A^c^
	p-value^d^ < 0∙001		p-value = 0∙70	
**RIF**	χ^2^ = 96∙7	LPA*** > MODS***> PSQ	χ^2^ = 16∙9	MODS** > PSQ
	p-value < 0∙001		p-value < 0∙001	
**MOX**	χ^2^ = 2∙0	N/A	χ^2^ = 0∙75	N/A
	p-value = 0∙37		p-value = 0∙69	
**OFX**	χ^2^ = 1∙6	N/A	χ^2^ = 1∙6	N/A
	p-value = 0∙44		p-value = 0∙46	
**AMK**	χ^2^ = 77∙9	PSQ*** > MODS** > LPA	χ^2^ = 2∙0	N/A
	p-value < 0∙001		p-value = 0∙37	
**KAN**	χ^2^ = 77∙9	PSQ*** > MODS** > LPA	χ^2^ = 16∙7	MODS* > LPA & PSQ
	p-value < 0∙001		p-value < 0∙001	
**CAP**	χ^2^ = 77∙9	PSQ*** > MODS** > LPA	χ^2^ = 1∙0	N/A
	p-value < 0∙001		p-value = 0∙61	

*p-value ≤ 0∙05

**p-value < 0∙01

***p-value < 0∙001

^a^Cochran’s Q test used to identify overall differences between assays for each drug.

^b^McNemar pairwise comparisons test used to which assays differed significantly for each drug.

^c^Pairwise comparisons were not performed if the Cochran’s Q test was not statistically significant (N/A).

^d^P-values were continuity corrected for multiple comparisons using Bonferroni correction.

With regards to the ability of the assays to detect resistance as defined by MGIT DST, among isolates that produced an interpretable result for all rapid tests, significant differences were evident only for the detection of RIF and KAN resistance. MODS detected significantly more RIF resistance compared to PSQ, and significantly more KAN resistance than both LPA and PSQ. All three tests had similar resistance detection capabilities for the remaining study drugs.

### Time to Result

**Fig 3:**
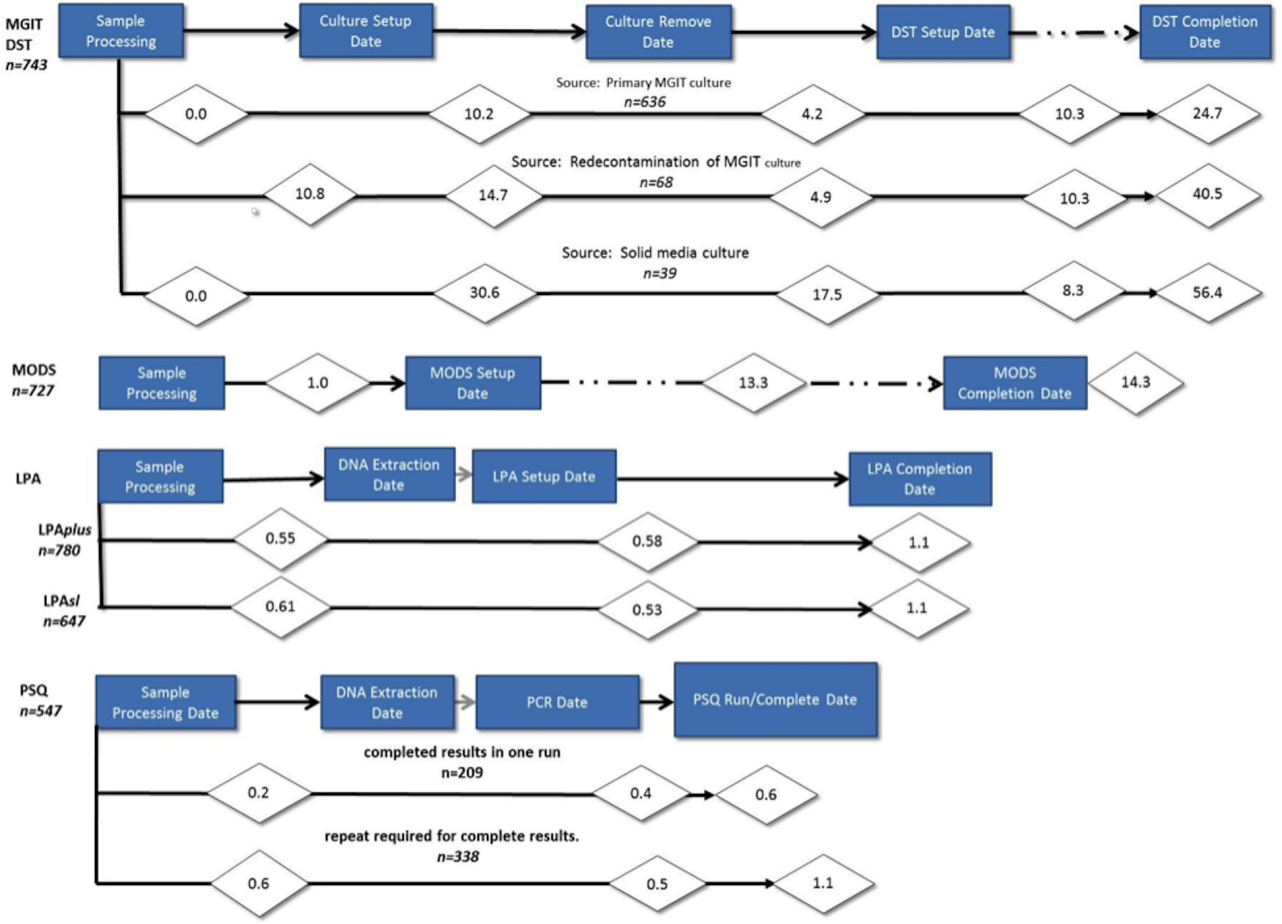
Time to Test Results (TTR). Criteria for inclusion in the time-to-result calculations: tests worked for all drugs tested on the first attempt unless otherwise noted. Only results from the culture positive pooled (P1) samples were used. For the growth-based assays MGIT DST and MODS, TTR was the period from sample processing through test completion. For the genotypic tests, TTR was sample processing through DNA extraction and PCR/test set up through completion, excluding time DNA was stored for batching or lab convenience.

The mean TTR for phenotypic DST originating from MGIT culture was 24.7 days, from decontaminated MGIT culture was 40.5 days, and from LJ was 56.4 days (Fig 3). For the MODS assay, the average TTR was 14.3 days, while the MTBDR*plus* and MTBDR*sl* LPAs had an average TTR of 1.1 days. PSQ results were grouped into samples that gave valid results for all targets in a single run and those which required repeat runs of the assay to obtain a valid result for all drugs. A single PSQ run was sufficient to yield a valid result for all targets in an average of 0.6 days. For those samples requiring a second PSQ run to produce valid results for all targets, it took an average of 1.1 days to produce valid results.

## Discussion

This study simultaneously assessed the performance of two DNA-based rapid diagnostic assays (LPA and PSQ) and one growth-based assay (MODS) to detect drug resistance in concentrated sputum specimens from 1,128 persons suspected of having M/XDR-TB. All three assays performed well among *Mtb* culture positive specimens for detection of INH, RIF, MOX, and OFX resistance despite 16% smear negative patients and 12% HIV co-infection. Our experimental design (where every sample received every test) allowed us to statistically investigate performance differences and determine that MTBDR*plus* outperformed both PSQ and MODS for INH and RIF in ability to obtain interpretable results, even though sensitivity and specificity did not appear to be different from PSQ and MODS. As described in the Methods section, only samples that gave interpretable results by both MGIT and the rapid test of interest were used to calculate sensitivity and specificity, resulting in variation in the number of comparisons available for each assay and each antibiotic.

However, in terms of the second-line drugs AMK, KAN, and CAP, it is clear that PSQ produced more interpretable results than the other two tests even though all tests exhibited only moderate sensitivity for detection of AMK and CAP (84%) and poor sensitivity for KAN resistance (50%).

Together, MTBDR*plus* and MTBDR*sl* LPAs comprise the only global, commercially available assays currently able to detect XDR-TB at the district hospital level or lower [14,31]. Our usage of the MTBDR*plus* version 2 is the first large-scale clinical evaluation of the updated assay and our findings confirm that version 2 has similar performance for direct detection of RIF resistance as version 1 (97%/98% vs. WHO pooled sensitivity/specificity 98%/98%) [32]. However, we demonstrated a substantially higher sensitivity for INH resistance detection than the pooled sensitivity of the version 1 assay of 94% (95%CI 91, 96) vs. 84% (95%CI 77, 90) reported by the WHO Expert Panel [11,13].

In our study, MTBDR*sl*, demonstrated substantially higher sensitivity for detection of MOX and OFX: 95% (95%CI: 91.8–97.6) compared to the WHO Expert Panel pooled sensitivity of 84% (95%CI: 61.9–91.0), but had comparable specificity of 99% vs. 97% reported by WHO [33]. Sensitivity of detection of AMK and CAP resistance were comparable to that documented by the WHO Expert Panel (87% vs. 86%) [33]. However, sensitivity for the detection of KAN resistance of 48% (95%CI: 38–58) was significantly below the WHO Expert Panel of 96% (95%CI: 67%-100%) [14]. This lower sensitivity was likely influenced by the high prevalence of *eis* promoter mutations at the Moldova study site [9], which are not detected by version 1.0 of the MTBDR*sl* assay. A new version of the assay including these *eis* promoter mutations, MTBDR*sl* version 2.0, is currently being evaluated, with a recent study finding an improved assay sensitivity of 86.4% in a multi-region study with 21.3% of injectable-resistant strains housing *eis* promoter mutations [34].

A major strength of the LPA assay is that it is currently commercially available in a kit form. Furthermore, while LPA is primarily a single sample test, it can be scaled-up to accommodate large numbers, and can produce results in a day. Limitations of LPA include the ratio of tests/interpretable results (S2 Table), the relatively high level skill required to run the assay (e.g. DNA processing), the comparatively high costs of the equipment compared to MODS, and the relative inflexibility of the assay to accommodate new mutations as knowledge of resistance-conferring mutations evolves.

In contrast to LPA, the major strength of the PSQ assay is its flexibility as an open sequencing technology, which allows for extensive post-processing analyses based on evolving knowledge, and its rapid adaptability to new mutations. The platform we evaluated in this study is one of the only XDR-TB diagnostics certified by Clinical Laboratory Improvement Amendments in the US, and this is the first large-scale clinical evaluation of the platform on direct sputum samples. The ability of PSQ to detect resistance to AMK, KAN, and CAP was superior compared to LPA and MODS, and was comparable to LPA for detection of INH, MOX, and OFX resistance, but had over twice the number of indeterminate results for the RIF resistance target—due mostly to amplification failures of the *rpoB* target. Given that both LPA and PSQ interrogate similar *rpoB* mutations, this suggests that further optimization of the PSQ *rpoB* amplification primers might improve performance.

Our previous studies of resistance-conferring mutations within *rrs* predicted that assays such as PSQ, capable of detecting the *rrs* 1401 mutation, should have approximately 88% sensitivity for detection of AMK and CAP resistance and 69% for KAN [9]. While reported sensitivities for AMK and CAP were both within 95% CI of our predictions (84%; 73–91% CI), reported sensitivities for KAN (50%; 41–60% CI) were lower than predicted. Preliminary, post study sequencing analysis of these isolates (data not shown) indicates that low KAN sensitivity was likely due to mutations within the *eis* promoter region that were not detected the PSQ assay. Future iterations of the platform will include detection of *eis* promoter and additional *rrs* mutations, which should increase sensitivity to AMK, KAN and CAP. Specificity of the assay for all targets was excellent and should increase confidence in the use of DNA-based diagnostics such as PSQ and LPA for ruling-in resistance to AMK, KAN and CAP. Limitations of the current PSQ platform include the need for high-level technical skills for handling of the complex, costly PSQ equipment, and a multi-step sequencing and analysis process. An integrated, tabletop PSQ platform is currently in development.

Our study is the first large evaluation of an XDR-TB MODS assay and we found it to be a highly sensitive and specific means of detecting INH, RIF, MOX, and OFX resistance direct from sputum and a moderately sensitive, highly specific means for detecting AMK and CAP resistance. MODS sensitivity for detection of KAN resistance was poor (62%), but MODS performed significantly better than both LPA and PSQ in terms of detecting KAN resistance, largely due to the fact that neither the LPA nor the PSQ assay included detection of *eis* mutations which are now well known to confer a significant proportion of phenotypic KAN resistance in *Mtb*, especially in places like Moldova where a large proportion of KAN resistance was observed in our study. Next generation versions of both the Hain LPA and PSQ assays now include *eis* mutations which should considerably improve the detection of phenotypic KAN resistance by these assays.

The reason for the low MODS sensitivity observed for the detection of KAN resistance was less clear. Our decision to use 5μg/ml in the MODS assay as the critical concentration for KAN resistance was made based on extensive breakpoint optimization studies [10], but this critical concentration is substantially higher than the WHO’s most recently recommended critical concentration for phenotypic KAN DST (2.5μg/ml) on MGIT960 [24]. Like MODS, MGIT DST is based on a liquid culture method, and this reliance upon different critical concentrations when using the two growth-based technologies could help explain the low sensitivity we observed. While there are reasons to support the use of different optimal critical concentrations between the MODS and MGIT960 (as the two technologies use different methods), our data suggests revising the MODS critical concentration for KAN downward towards the concentration recommended for MGIT960.

The major strengths of the MODS assay is that it is a rapid (10 days quicker than MGIT DST), cheap, non-commercial assay that can be used to detect M/XDR-TB directly from processed sputum with inexpensive, readily purchased supplies or commercial kits [19,35–37]. Concerns over biohazard risk associated with daily readings of MODS have been eliminated by modifying the procedure to seal plates after inoculation to contain the infectious organisms. Finally, additional implementation considerations include MODS's logistical requirements, which could limit the assays utility in high-throughput environments.

In conclusion, we have demonstrated that three existing technologies have very good to excellent sensitivity and specificity for the detection of resistance to the highest priority antibiotics used for TB: RIF, INH, MOX, and OFX. The observed differences in test performance by antibiotic may be the result of differences in primers used in the two gene based tests and concentrations of antibiotics used as cut off points in the two growth based tests. Additionally, each technology has limitations which likely confine them to use at the district, sub-district hospital level in their current forms.

Rapid initiation of treatment with a set of drugs to which *Mtb* is susceptible remains the most successful intervention in preventing the spread of M/XDR-TB and improving patient outcomes. Each of the three rapid DST assays we examined have major advantages over the MGIT in terms of saving weeks to months for the identification of drugs to which the organism is resistant while maintaining acceptable test performance. In particular, the very high specificity of detection of drug resistance means that clinicians can use the results of these rapid tests to avoid the use of toxic drugs to which the infecting organism is resistant and develop treatment regiments that have a higher likelihood of yielding a successful outcome. The availability of this information within a few hours of testing allows clinicians to quickly find effective treatment regimens and put a stop to the transmission of drug-resistant TB. These data suggest that the current recommendation for the initial treatment of active TB in areas of high rates of drug resistance can be improved by the use of rapid DST early in the treatment regimen. The very high specificity of the rapid identification of resistance to the drugs studied means that when resistance is identified one can to avoid the use of drugs that will not be efficacious but may increase toxicity. This strategy may decrease the infectious period, reduce treatment failure and may decrease defaults while reducing costs.

## Supporting Information

S1 Dataset GCDD Dataset. (TXT)

S1 Table Rapid Assay Performance. Agreement between three rapid tests and reference standard MGIT for detection of resistance for isoniazid (INH), rifampin (RIF), moxifloxacin (MOX), ofloxacin (OFX), amikacin (AMK), kanamycin (KAN), and capreomycin (CAP) Mtb culture positive specimens.
(DOCX)

S2 Table Interpretable Results. Proportion of Mtb culture positive specimens for which interpretable results were produced by three diagnostic platforms with the ability to detect resistance to isoniazid (INH), rifampin (RIF), moxifloxacin (MOX), ofloxacin (OFX), amikacin (AMK), kanamycin (KAN), and capreomycin (CAP).
(DOCX)

S1 Text GCDD Contributors. (DOCX)
