# Supplementary material for: Performance Comparison of Three Rapid Tests for the Diagnosis of Drug-Resistant Tuberculosis
Source: PLoS One. 2015 Aug 31;10(8):e0136861. doi: 10.1371/journal.pone.0136861 (PMC4556461; doi:10.1371/journal.pone.0136861)
Supplement: S1 Table — Agreement between three rapid tests and reference standard MGIT for detection of resistance for isoniazid (INH), rifampin (RIF), moxifloxacin (MOX), ofloxacin (OFX), amikacin (AMK), kanamycin (KAN), and capreomycin (CAP) Mtb culture positive specimens. (DOCX) [file pone.0136861.s002.docx]

Table S1. Rapid Assay Performance. Agreement between three rapid tests and reference standard MGIT for detection of resistance for isoniazid (INH), rifampin (RIF), moxifloxacin (MOX), ofloxacin (OFX), amikacin (AMK), kanamycin (KAN), and capreomycin (CAP) *Mtb* culture positive specimens.

|  |  | Sensitivity (95% CI) | Specificity (95% CI) | PPV (95% CI) | NPV (95% CI) | LR+ (95% CI) | LR- (95% CI) | Agreement (95% CI) |
| --- | --- | --- | --- | --- | --- | --- | --- | --- |
| INH | LPA (n=790) | 0.939 (0.914, 0.958) | 0.996 (0.977, 1.000) | 0.998 (0.987, 1.000) | 0.900 (0.860, 0.930) | 264 (37, 1867) | 0.061 (0.043, 0.086) | 0.959 (0.943, 0.972) |
|  | MODS (n=729) | 0.970 (0.950, 0.982) | 0.987 (0.959, 0.997) | 0.994 (0.981, 0.998) | 0.938 (0.897, 0.964) | 74 (24, 228) | 0.030 (0.018, 0.050) | 0.975 (0.960, 0.985) |
|  | PSQ (n=779) | 0.953 (0.930, 0.968) | 0.961 (0.925, 0.981) | 0.983 (0.967, 0.992) | 0.895 (0.849, 0.929) | 24 (13, 46) | 0.049 (0.034, 0.072) | 0.955 (0.937, 0.968) |
| RIF | LPA (n=809) | 0.967 (0.945, 0.980) | 0.979 (0.955, 0.991) | 0.985 (0.968, 0.993) | 0.953 (0.923, 0.972) | 46 (22, 95) | 0.034 (0.021, 0.055) | 0.972 (0.957, 0.981) |
|  | MODS (n=729) | 0.996 (0.983, 0.999) | 0.978 (0.950, 0.991) | 0.987 (0.971, 0.995) | 0.992 (0.970, 0.999) | 45 (20, 99) | 0.004 (0.001, 0.018) | 0.989 (0.978, 0.995) |
|  | PSQ (n=661) | 0.938 (0.911, 0.958) | 0.991 (0.964, 0.998) | 0.995 (0.981, 0.999) | 0.891 (0.843, 0.925) | 104 (26, 414) | 0.062 (0.043, 0.089) | 0.956 (0.937, 0.970) |
| MOX | LPA (n=742) | 0.955 (0.918, 0.976) | 0.990 (0.975, 0.996) | 0.979 (0.949, 0.992) | 0.978 (0.960, 0.989) | 95 (40, 228) | 0.046 (0.026, 0.081) | 0.978 (0.964, 0.987) |
|  | MODS (n=727) | 0.978 (0.951, 0.991) | 0.971 (0.950, 0.984) | 0.954 (0.921, 0.974) | 0.986 (0.969, 0.994) | 34 (20, 58) | 0.022 (0.010, 0.049) | 0.974 (0.959, 0.984) |
|  | PSQ (n=744) | 0.938 (0.900, 0.962) | 0.983 (0.966, 0.992) | 0.970 (0.939, 0.986) | 0.965 (0.943, 0.979) | 55 (28, 110) | 0.064 (0.040, 0.101) | 0.966 (0.950, 0.978) |
| OFX | LPA (n=742) | 0.959 (0.924, 0.979) | 0.994 (0.981, 0.998) | 0.987 (0.960, 0.997) | 0.980 (0.963, 0.990) | 159 (52, 492) | 0.041 (0.022, 0.076) | 0.982 (0.969, 0.990) |
|  | MODS (n=729) | 0.982 (0.957, 0.993) | 0.980 (0.961, 0.990) | 0.969 (0.939, 0.985) | 0.989 (0.972, 0.996) | 49 (25, 93) | 0.018 (0.008, 0.043) | 0.981 (0.967, 0.989) |
|  | PSQ (n=745) | 0.942 (0.905, 0.965) | 0.991 (0.977, 0.997) | 0.985 (0.959, 0.995) | 0.967 (0.946, 0.980) | 111 (42, 294) | 0.059 (0.036, 0.094) | 0.973 (0.958, 0.983) |
| AMK | LPA (n=672) | 0.868 (0.740, 0.941) | 1.000 (0.992, 1.000) | 1.000 (0.904, 1.000) | 0.989 (0.976, 0.995) | - | 0.132 (0.066, 0.263) | 0.990 (0.978, 0.995) |
|  | MODS (n=729) | 0.900 (0.799, 0.955) | 0.995 (0.986, 0.999) | 0.955 (0.864, 0.988) | 0.989 (0.977, 0.995) | 198 (64, 613) | 0.100 (0.050, 0.203) | 0.986 (0.974, 0.993) |
|  | PSQ (n=801) | 0.836 (0.727, 0.909) | 0.993 (0.983, 0.997) | 0.924 (0.825, 0.972) | 0.984 (0.971, 0.991) | 122 (50, 293) | 0.166 (0.099, 0.278) | 0.979 (0.966, 0.987) |
| KAN | LPA (n=672) | 0.479 (0.377, 0.583) | 1.000 (0.992, 1.000) | 1.000 (0.904, 1.000) | 0.920 (0.895, 0.940) | - | 0.521 (0.430, 0.631) | 0.926 (0.902, 0.944) |
|  | MODS (n=729) | 0.619 (0.524, 0.705) | 0.998 (0.989, 1.000) | 0.986 (0.917, 0.999) | 0.931 (0.908, 0.949) | 378 (53, 2693) | 0.382 (0.304, 0.481) | 0.937 (0.916, 0.953) |
|  | PSQ (n=801) | 0.504 (0.412, 0.596) | 0.993 (0.982, 0.997) | 0.924 (0.825, 0.972) | 0.918 (0.896, 0.937) | 69 (28, 167) | 0.500 (0.417, 0.598) | 0.919 (0.897, 0.936) |
| CAP | LPA (n=672) | 0.863 (0.731, 0.938) | 0.997 (0.987, 0.999) | 0.957 (0.840, 0.992) | 0.989 (0.976, 0.995) | 268 (67, 1073) | 0.138 (0.069, 0.274) | 0.987 (0.974, 0.993) |
|  | MODS (n=729) | 0.851 (0.738, 0.922) | 0.994 (0.983, 0.998) | 0.934 (0.833, 0.979) | 0.985 (0.972, 0.992) | 141 (53, 376) | 0.150 (0.085, 0.266) | 0.981 (0.967, 0.989) |
|  | PSQ (n=801) | 0.843 (0.732, 0.915) | 0.990 (0.979, 0.996) | 0.894 (0.788, 0.953) | 0.985 (0.973, 0.992) | 88 (42, 185) | 0.159 (0.092, 0.273) | 0.978 (0.964, 0.986) |

Sensitivity was calculated as TP/(TP+FN), specificity as TN/FP+TN), PPV as TP/(TP+FP), NPV as TN/(TN+FN), LR+ as sensitivity/(1-specificity), LR- as (1-sensitivity)/specificity, and accuracy as (TP +TN)/(TN+FN+FP+TP). Proportion confidence intervals were calculated using the Wald score method with continuity correction.
