# Supplementary material for: Performance Comparison of Three Rapid Tests for the Diagnosis of Drug-Resistant Tuberculosis
Source: PLoS One. 2015 Aug 31;10(8):e0136861. doi: 10.1371/journal.pone.0136861 (PMC4556461; doi:10.1371/journal.pone.0136861)
Supplement: S2 Table — Proportion of Mtb culture positive specimens for which interpretable results were produced by three diagnostic platforms with the ability to detect resistance to isoniazid (INH), rifampin (RIF), moxifloxacin (MOX), ofloxacin (OFX), amikacin (AMK), kanamycin (KAN), and capreomycin (CAP). (DOCX) [file pone.0136861.s003.docx]

Table S2. Interpretable Results. Proportion of *Mtb* culture positive specimens for which interpretable results were produced by three diagnostic platforms with the ability to detect resistance to isoniazid (INH), rifampin (RIF), moxifloxacin (MOX), ofloxacin (OFX), amikacin (AMK), kanamycin (KAN), and capreomycin (CAP).

|  | **LPA**  (n/%) | | **PSQ**  (n/%) | | **MODS**  (n/%) | |
| --- | --- | --- | --- | --- | --- | --- |
| **INH** | 793/914 | 87% | 786/914 | 86% | 730/914 | 80% |
| **RIF** | 812/914 | 89% | 667/914 | 73% | 730/914 | 80% |
| **MOX** | 745/914 | 82% | 749/914 | 82% | 730/914 | 80% |
| **OFX** | 745/914 | 82% | 749/914 | 82% | 730/914 | 80% |
| **AMK** | 675/914 | 74% | 806/914 | 88% | 730/914 | 80% |
| **KAN** | 675/914 | 74% | 806/914 | 88% | 730/914 | 80% |
| **CAP** | 675/914 | 74% | 806/914 | 88% | 730/914 | 80% |

LPA=line probe assay, PSQ=pyrosequencing, MODS= Microscopic Observation of Drug Susceptibility
