# Supplementary material for: Performance Comparison of Three Rapid Tests for the Diagnosis of Drug-Resistant Tuberculosis
Source: PLoS One. 2015 Aug 31;10(8):e0136861. doi: 10.1371/journal.pone.0136861 (PMC4556461; doi:10.1371/journal.pone.0136861)
Supplement: S1 Text — (DOCX) [file pone.0136861.s004.docx]

**S1 Text. GCDD Contributors**

We thank the GCDD teams for their participation in this study: At P.D. Hinduja Hospital and Medical Research Center, Mumbai, India: Clinical Investigator Zarir Udwadia, M.D., FRCP, FCCP; Clinical and Laboratory Project Coordinator Kanchan Ajbani, Ph.D.; Consultant Microbiologist Anjali Shetty, M.D.; Laboratory Technician Maria Jose, and Laboratory Research Scientist V.P. Vineeth. At the Institute of Phthisiopneumology, Chisinau, Moldova: Clinical Investigators Sofia Alexandru, M.D., Victor Botnaru, M.D., Ph.D., and Dumitru Chesov; Microbiologist Ecaterina Noroc, M.D.; Laboratory Research Scientists Victor Lesan, M.D., Nicolae Moraru, M.D., Ph.D., Elena Romancenco, Eugenia Rusu, Najdeda Turcan, and Svetlana Verejan; Researcher Alexandru Buga, M.D., and Administrative/Scientific Liaison Viorel Soltan, M.P.H., M.B.A., Ph.D. At Stellenbosch University, Cape Town, South Africa: Scientist Rob Warren and Study Investigator Lynthia V. Paul, Ph.D. At Port Elizabeth, the National Health Laboratory Service: Laboratory Manager Cindy Hayes; Study Nurse Sister Ann Mkosana; Medical Technologist Sabine Klages. Additionally, we would like to acknowledge the work on the MODS assay by David Moore, M.D., Jorge Coronel, M.S. and Luz Caviedes, affiliated with the Imperial College London in Lima, Peru. In addition, the contribution of David Engelthaler, M.S. from the Translational Genomics Research Institute (T-Gen), Phoenix, Arizona for his involvement in genome sequencing and data analysis. Finally, we acknowledge at the University of California at San Diego, California the contribution of Elisea Avalos, M.P.H., Theodore Ganiats, M.D., Janice Kaping, M.S., C.L.S, Lisa Kim, M.P.H., Marisa Magana, M.D., Daniel Park, M.D., Mark Pettigrove, M.P.H., and Daniel Schatzle for providing data, laboratory, analytic, and administrative support.
